# Selective maintenance of *Drosophila *tandemly arranged duplicated genes during evolution

**DOI:** 10.1186/gb-2008-9-12-r176

**Published:** 2008-12-16

**Authors:** Carlos Quijano, Pavel Tomancak, Jesus Lopez-Marti, Mikita Suyama, Peer Bork, Marco Milan, David Torrents, Miguel Manzanares

**Affiliations:** 1 Instituto de Investigaciones Biomédicas CSIC-UAM, Arturo Duperier 4, 28029 Madrid, Spain; 2 Barcelona Supercomputing Center, Jordi Girona 31, 08034 Barcelona, Spain; 3 Max Planck Institute of Molecular Cell Biology and Genetics, Pfotenhauerstrasse 108, D-01307 Dresden, Germany; 4 European Molecular Biology Laboratory, Meyerhofstrasse 1, 69117 Heidelberg, Germany; 5 ICREA and Institute for Research in Biomedicine (IRB), Parc Científic de Barcelona, Josep Samitier 1-5, 08028 Barcelona, Spain; 6 ICREA and Barcelona Supercomputing Center, Jordi Girona 31, 08034 Barcelona, Spain; 7 Centro Nacional de Investigaciones Cardiovasculares (CNIC), Melchor Fernandez Almagro 3, 28029 Madrid, Spain; 8 Current address: Center for Genomic Medicine, Kyoto University Graduate School of Medicine, Kyoto 606-8501 Japan

## Abstract

**Background:**

The physical organization and chromosomal localization of genes within genomes is known to play an important role in their function. Most genes arise by duplication and move along the genome by random shuffling of DNA segments. Higher order structuring of the genome occurs in eukaryotes, where groups of physically linked genes are co-expressed. However, the contribution of gene duplication to gene order has not been analyzed in detail, as it is believed that co-expression due to recent duplicates would obscure other domains of co-expression.

**Results:**

We have catalogued ordered duplicated genes in *Drosophila melanogaster*, and found that one in five of all genes is organized as tandem arrays. Furthermore, among arrays that have been spatially conserved over longer periods than would be expected on the basis of random shuffling, a disproportionate number contain genes encoding developmental regulators. Using *in situ *gene expression data for more than half of the *Drosophila *genome, we find that genes in these conserved clusters are co-expressed to a much higher extent than other duplicated genes.

**Conclusions:**

These results reveal the existence of functional constraints in insects that retain copies of genes encoding developmental and regulatory proteins as neighbors, allowing their co-expression. This co-expression may be the result of shared *cis*-regulatory elements or a shared need for a specific chromatin structure. Our results highlight the association between genome architecture and the gene regulatory networks involved in the construction of the body plan.

## Abstract 2

Genes occurring in conserved, tandemly-arrayed clusters in Drosophila melanogaster are co-expressed to a much higher extent than other duplicated genes.

## Background

The simple idea that the functionality of eukaryotic genomes is determined solely by the content of genes and their regulatory regions has been gradually replaced by a more complex view, which recognizes a crucial role for the way in which these functional elements are distributed and organized. The discovery that some groups of genes with particular organizations (normally neighboring genes) have been conserved over long periods confirms that, at least in some cases, proximity between genes is essential for their functionality. The fruitfly *Drosophila melanogaster *contains several examples of duplicated genes arranged in such a fashion and that are involved in embryonic patterning; these include the *en-inv *[1], *ey-toy *[2] and *eyg-toe *[3] pairs, the *achaete-scute *[4], *Enhancer-of-split *[5] and *iroquois *clusters [6], and most significantly the *Antennapedia *and *Bithorax Hox *complexes [7], whose genomic organization has been conserved since the appearance of metazoans. The identification of substantial overlap in the expression patterns between genes within these groups suggests that these arrangements might be first fixed and subsequently maintained by the need for certain shared regulatory regions.

Beyond these specific examples, a number of large-scale computational studies have attempted to detect and measure the level of gene organization within eukaryotic genomes. These analyses searched for significant correlation between gene order and co-expression, under the assumption that neighboring genes will be expressed in a concerted way (for a review, see [8]). However, the results of these studies, normally consisting of rather weak correlation signals, are insufficient to provide an understanding of the overall gene organization in eukaryotic genomes. This is the case not only when comparing gene order with co-expression, but also when comparing groups of genes belonging to different functional classes or involved in the same process or pathway [9-11]. In contrast to prokaryotes, where functionally related and co-expressed neighboring genes (mostly arranged in operons) are abundant and easily identified, eukaryotic genomes present an apparently much more complex organization, in which genes with no obviously ordered distribution predominate and co-exist with a smaller class of clustered, co-expressed genes. An important limitation of previous analyses is that, because of their global nature, they were unable to identify which genes require a particular genomic arrangement for their function and are, therefore, directly responsible for the detected correlation signal. Furthermore, many large-scale studies have deliberately not considered duplicated genes in order to exclude a disproportionate co-expression signal due to recently duplicated genes [12-14], despite the fact that most co-expressed neighboring genes in eukaryotes appear to have arisen by gene duplication. In fact, the identification of these clusters of duplicated genes underlines the importance of gene organization in eukaryotic genomes [8], and provides important information about how genes evolve after their duplication.

Here, we have combined computational and experimental approaches to identify and characterize all detectable duplicated genes that have been conserved in close proximity in the *Drosophila *genome. Through analysis of available *in situ *expression data we have also evaluated the expression pattern of the detected cases in order to determine their level of co-expression. We found that a number of duplicated genes have been retained as tandems over a longer period than would be expected in the absence of selective constraints, and that this gene set is enriched in genes involved in developmental processes as well as those encoding transcriptional regulators. Furthermore, we show that these ancient tandem duplicates show a higher level of co-expression than other genes, even recently duplicated tandem pairs.

## Results and discussion

### Identification and organization of duplicated genes in *Drosophila*

As a first step towards the identification and characterization of duplicated genes conserved in proximity, we evaluated how duplicated gene pairs are generally distributed along the fly genome. For this, we first identified duplicated gene pairs (paralogues) by comparing *Drosophila *protein products (see Materials and methods) and then evaluated the distance separating the duplicate genes on the same chromosome (expressed as the number of 'intervening non-paralogous genes': i-genes). This analysis showed that a predominant fraction of paralogous genes have zero or few intervening genes. The number of paralogous pairs decreases exponentially thereafter as the number of intervening genes increases (Additional data file 1). This distribution probably reflects the abundance of recently duplicated gene copies, which are still arranged as they were formed, that is, in tandem.

### Genes originated by tandem duplication separate with time in a non-linear fashion

**Figure 1:**
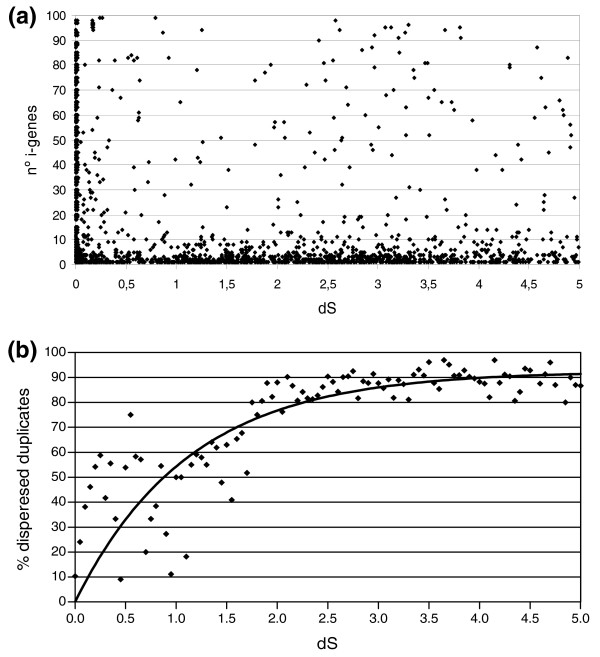
**Genomic and temporal distribution of duplicated genes in *D. melanogaster***. **(a) **The distance between duplicates does not increase sequentially with time, as estimated by dS values. The majority of gene pairs are either very near or far apart. The most frequent profiles for duplicated genes are (but not restricted to) consecutive (i-genes approximately 0) or recently (dS approximately 0) duplicated genes or both. Only pairs separated by up to 100 intervening genes and with dS < 5 are shown. **(b) **The proportion of pairs of duplicated genes that have separated increases over time, reaching a point where more than 90% of all duplicated genes are not physically linked. For example, there are 240 linked pairs in the dS 0-0.5 range, while there are only 19 for dS 4.5-5.0. The best fit exponential distribution that reaches a plateau at 92.51% is shown as a solid line.

If we take as our null hypothesis that duplicated gene pairs will gradually separate over time as the result of random genome reorganization events, such as inversions and translocations [15], we can predict that the physical distance, or the number of i-genes, separating duplicate genes that originated in tandem should gradually increase with time. And indeed, this general tendency is observed when we compare the number of i-genes and the relative age (inferred from the degree of neutral sequence divergence, dS) for each duplicated gene pair; the number of gene copies separated by many i-genes (>100) appears to be higher for older duplicated pairs, indicating that most duplicated genes are not under selection pressure to remain in proximity and can separate over time (not shown). However, the physical separation between the copies does not appear to be gradual, because we do not observe a linear correlation between genetic distance and age, but rather an all-or-nothing phenomenon, whereby duplicate genes are either co-localized or are dispersed at distant locations in the genome (Figure 1a). By comparing the exon-intron structures of duplicated pairs, we further discarded possible biases and ruled out that this pattern could be due to a massive presence of retrogenes in *Drosophila *[16] (see Materials and methods). This pattern of gene separation, which implies profound remodeling of the genome, is consistent with the extreme degree of chromosomal rearrangement found in *Drosophila *[17-19], rather than with a predominance of micro-inversions and small insertions, which would move and shuffle genes gradually within chromosomes.

### A high degree of gene duplication and arrangement of duplicate genes in tandem arrays is found in the *Drosophila *genome

To distinguish tandem from dispersed duplicates, we needed to determine at what level of sequence divergence (that is, at which relative age) we can expect any two duplicated genes to have separated from each other. To answer this, we first classified all detectable gene copies into two groups: tandemly arrayed duplicated genes (TDGs) and dispersed duplicates. TDGs were conservatively defined as those separated by 10 i-genes or fewer, based on a statistical comparison of the actual distribution of duplicated gene pairs with 10,000 distributions of randomly arranged pairs (Additional data file 2). Using these parameters, we found that of the 8,664 genes detected as duplicates in *D. melanogaster *(59% of all genes), 2,952 are organized in tandem, in agreement with previous estimates [11]. This represents one in three (34%) of all duplicated genes, and one in five (20%) of the whole gene set, a figure only slightly higher than that observed in mammals and plants [20,21]. When we explored how duplicated genes tend to separate with time, we found that the proportion of gene duplicates that are dispersed increases linearly with the level of neutral sequence divergence, that is, with time. This relationship reached a maximum (roughly between dS values of 3 and 4), beyond which it appears that practically all duplicated pairs that can freely separate from each other have done so and remain apart (Figure 1b). These data follow an exponential distribution that reaches a plateau at 92.51% (*p*-value < 0.05, when compared to a distribution assymptoting at 100%), from which we can conclude that there is a fraction of duplicated genes that do not separate over time. The same behavior was observed using, instead of dS, dN (number of non-synonymous substitutions per site) as an estimator of the relative age of the duplicates, which is expected to be more inaccurate than dS, as it depends on levels of purifying selection and these, on gene function (data not shown). This observation suggests that some of those gene pairs that show high levels of sequence divergence and still remain as neighbors could have been retained in tandem due to selective constraints. On the other hand, this behavior could also simply reflect a passive and neutral retention of duplicates in tandem over long time periods. As a way to distinguish between both possibilities, we examined if there is any functional difference in this set of genes that could derive from a selective retention of certain classes of tandem duplicates.

### Evolutionarily conserved tandem duplicates are significantly enriched for developmental and regulatory genes

Gene Ontology (GO) analysis [22] reveals that the set of TDGs with a high degree of sequence divergence (2,012 genes with dS > 4), likely representing 'old' linked genes, is significantly enriched in genes encoding functions related to embryonic development and transcriptional regulation when compared to dispersed duplicated genes (Additional data file 3). This association was not observed with younger gene duplicates (1,523 genes with dS < 4), and even more, a high number of these functions were observed among the under-represented GO terms for TDGs with dS values between 0 and 2. This finding suggests that developmental and regulatory genes are overrepresented among conserved linked gene copies, and that the known examples previously described [1-7] are not just anecdotal and known because of a biased sampling from the literature.

However, the use of dS values beyond saturation (dS > 2 or 3) should still be treated with great caution, despite being used at large scale and to detect a general behavior of genes. For this reason, we next used an alternative approach to accurately classify and select a collection of 'old' gene duplicates, and re-evaluate them at the level of potential functional enrichments. To do so, we obtained a collection of gene duplicates through a phylogenetic approach, searching for TDGs that we are certain have been conserved as such during the independent evolution of fly and mosquito since their divergence at least 250 million years ago [23]. This new set, although limited in size, is expected to be more reliable and to avoid the potential problems associated with the calculation of the neutral divergence (dS) of 'old' duplicates [24]. To obtain this new collection of conserved neighboring gene duplicates, we first applied the same procedure described above to identify and classify duplicate genes in the genome of the mosquito *Anopheles gambiae*. Compared to the 2,952 TDGs identified in *D. melanogaster*, we found 2,637 in mosquito. We then compared the TDG sets from *D. melanogaster *and *A. gambiae *and defined TDGs as evolutionarily conserved using orthologous duplicated genes that are arranged in tandem in both species and that fit a phylogenetic model consistent with the existence of the TDG group in a common ancestor (see Materials and methods). In this way, we defined the set of conserved TDGs, comprising 400 genes, grouped in 154 tandem arrays (the majority of which contained 2 or 3 duplicate genes; see Materials and methods). Consistent with our previous analysis (Figure 1b), more than 95% of these TDGs conserved between *A. gambiae *and *D. melanogaster *have gene duplicates with dS values > 2. This confirms that we are in fact dealing with a collection of gene duplicates with ranges of ages where nearly all duplicates are expected to be separated.

In order to further confirm that this set truly represents TDGs conserved throughout the dipteran lineage, we additionally checked for their presence in two other drosophilid genomes, *D. pseudoobscura *and *D. virilis*, which have divergence times from *D. melanogaster *of 27 and 40 million years ago, respectively. A minimum quality of genome assembly is crucial for a correct estimation of TDGs, and prohibited the inclusion in our analysis of other non-dipteran insect species with fragmentary genome assemblies [25,26]. We found that out of the 154 TDG arrays conserved between *D. melanogaster *and *A. gambiae*, 131 (85.1%) are also present in *D. pseudoobscura *and 122 (79.2%) in *D. virilis*. To determine whether or not this degree of conservation with other drosophilids can be explained by random processes of retention or loss of tandem duplicates, we analyzed the organization of 526 pairs of tandemly duplicated genes from *D. melanogaster *that are not conserved with *A. gambiae *in the genome of *D. pseudoobscura*. In order to ensure that the absence of conservation in this comparison is due to a separation or loss of duplicates in *D. pseudoobscura *and not to *D. melanogaster*-specific duplications, we did not count TDGs for which we could find only one or no orthologues in *D. pseudoobscura *or *D. virilis*. This gave us a group of 398 TDGs, of which 305 (76.6%) are conserved in *D. pseudoobscura*, showing that those TDGs that have been formed before the split of *A. gambiae *and *D. melanogaster *and have been conserved together since then have a higher probability to be also in tandem in a different drosophila than those TDGs of more recent evolutionary origin (two-tailed Fisher's exact test; *p*-value < 0.05).

Of the conserved tandem arrays between fly and mosquito, 33% (132 genes) are included in syntenic regions where gene order is conserved between *D. melanogaster *and *A. gambiae *[27]. This figure coincides with the overall percentage of *D. melanogaster*-*A. gambiae *orthologues remaining in synteny [25], showing that the conserved TDGs cannot, therefore, be explained by synteny alone.

**Figure 2:**
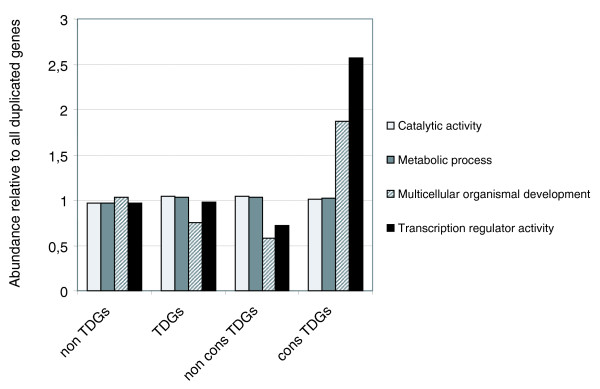
**Evolutionarily conserved TDGs are enriched in developmental and transcription factor genes**. The graph shows the ratio of the abundance of the listed GO categories in the different subsets of duplicated genes to the abundance among all duplicated genes. A value of 1 indicates that the abundance in a subset is comparable to that in the whole set. The conserved TDG subset is enriched in genes under the categories 'multicellular organismal developmental' and 'transcription factor activity'. *p*-values for individual children GO terms of these categories found to be overrepresented among conserved TDGs are all < 0.05 (Additional data file 5). Abbreviations: non TDGs, duplicated genes that are not arranged in tandem; TDGs, duplicated genes that are arranged in tandem; non cons TDGs, tandem duplicates that are not conserved in *A. gambiae*; cons TDGs, tandem duplicates that are conserved between *D. melanogaster *and *A. gambiae*.

We compared the GO distributions of TDGs and dispersed duplicates, and of conserved TDGs versus dispersed duplicates and non-conserved TDGs. We did not find significant differences in the distribution of functional categories between TDGs and dispersed duplicates (Additional data file 4). However, as with the constrained TDG set defined by neutral divergence criteria, the conserved TDG set is enriched in developmental and transcription factor genes in comparison with both the dispersed duplicates (21 out of 30 overrepresented GO terms with *p*-value < 0.05) and the non-conserved TDGs (30 out of 49 overrepresented GO terms with *p*-value < 0.05 (Additional data file 5)). To confirm that this trend held for all genes categorized as developmental or transcriptional regulators, we compared the abundance of genes annotated with four higher-level GO terms in each duplicate gene set relative to their abundance in the whole collection of duplicated genes (Figure 2; Additional data file 6). As expected, the relative abundance of genes annotated with 'catalytic activity' [GO:0003824] and 'metabolic process' [GO:0008152] was similar among all sets. In contrast, genes in the 'multicellular organismal developmental' [GO:0007275] and 'transcriptional regulator activity' [GO:0030528] categories were notably more abundant in the conserved TDG set. We observed these same trends when we removed from the conserved TDG set those genes that are located in syntenic regions (see above; data not shown). This further shows that the functional enrichment observed is not due to a fraction of TDGs being maintained as such because of evolutionary conservation of larger regions of the chromosome with conserved gene order.

To validate this observation, we repeated the analysis using, instead of GO categories, gene sets defined independently by Nelson and co-workers for another purpose [28]. These gene sets are 'complex' (genes with high regulatory complexity), 'HK' (house-keeping) and CDY (single genes in *C. elegans*, *D. melanogaster *and yeast). In this case, we observed a relative enrichment of 'complex' genes in the conserved TDG set (Additional data files 6 and 7). We can therefore affirm that certain classes of duplicated gene, mostly 'trans-dev' (developmental transcriptional factor) genes [29], are preferentially retained over evolutionary time in a tandem organization after duplication. Thus, the conservation of TDGs requires an explanation involving evolutionary forces that favor certain functions of the duplicated genes, and not the neutral drift of genome re-ordering.

### Evolutionarily conserved tandem duplicates are highly co-expressed

Considering our previous results, the conservation of tandem duplicates could be explained by the existence of shared *cis*-regulatory elements, making their separation deleterious for the organism and, therefore, less probable or impossible to fix in the population [30,31]. A prediction of this scenario is that conserved TDGs would be more likely to be co-expressed in time and space than other duplicate pairs. To test this hypothesis, we examined the database of gene expression patterns during embryogenesis in *D. melanogaster*, which to date encompasses the expression, by whole mount *in situ *hybridization, of nearly half the genome [32]. While these data are certainly scarcer than expression profiles based on DNA microarrays, they are much more information-rich, and thus a valuable complement to other studies [33]. For those gene clusters for which *in situ *patterns were available, two or more genes that share a characteristic expression domain in the embryo were scored as positive for co-expression. Maternal or ubiquitous expression was not considered as evidence for co-expression (see Materials and methods). In total, we scored the expression of 1,963 genes (Table 1).

**Table 1:** Number of groups and genes that show co-expression in the *D. melanogaster *embryo

	Number of groups* (genes^†^)	Number of co-expressing groups^‡ ^(genes^†^)	Percentage of co-expressing groups (genes^†^)
Conserved TDGs	52 (118)	19 (43)	36.5 (36.4)
Conserved non-TDGs^§^	179 (578)	38 (89)	21.3 (15.4)
Conserved neighbors^¶^	198 (716)	37 (107)	18.7 (14.9)

*Total number of groups where two or more genes have a reported *in situ *analysis. ^†^Number of genes that have been analyzed by *in situ*. ^‡^Number of groups where two or more genes show co-expression in at least one domain of the embryo. ^§^Groups of duplicated genes that are not arranged in tandem and that have one-to-one orthologues in *A. gambiae*. ^¶^Groups of genes that are located in syntenic regions between *D. melanogaster *and *A. gambiae*, that have one-to-one orthologues in *A. gambiae*, and that are not tandem duplicates.

Of the 154 evolutionarily conserved TDG clusters, *in situ *hybridization evidence was available for 52, and of these, 19 showed co-expression (36.6%; Additional data file 8). We also examined expression data for 179 dispersed duplicate gene pairs for which clear orthologues exist for both genes in *A. gambiae*. Of these groups, co-expression was found for 38 (21.3%). This analysis thus shows that tandemly arrayed duplicated genes that have been conserved in proximity since the divergence of *D. melanogaster *and *A. gambiae *are more likely to share a characteristic expression pattern in the early embryo than other duplicated genes (Chi squared; *p*-value < 0.05).

We next assessed whether co-expression was simply an effect of both genes being in the same genomic location [11,34]. Of 198 groups of genes examined that are not related by duplication but are linked in both *D. melanogaster *and *A. gambiae *(conserved neighbors), we found evidence of co-expression for 37, or 18.7% (Additional data file 9). Comparison with the figure of 36.6% for the evolutionarily conserved TDG clusters demonstrates that co-expression of conserved TDGs cannot be explained by being located in broader co-expression domains of the genome (Chi squared; *p*-value = 0.01). We are aware that this analysis is limited by the number of cases examined (Additional data file 10) and also by the fact that we can only use positive evidence, since two genes that are not co-expressed in the embryo may be so at later stages. We nevertheless have confidence in the results because of restrictive criteria use in the analysis, which would tend to underestimate the number of co-expressed conserved TDGs.

**Figure 3:**
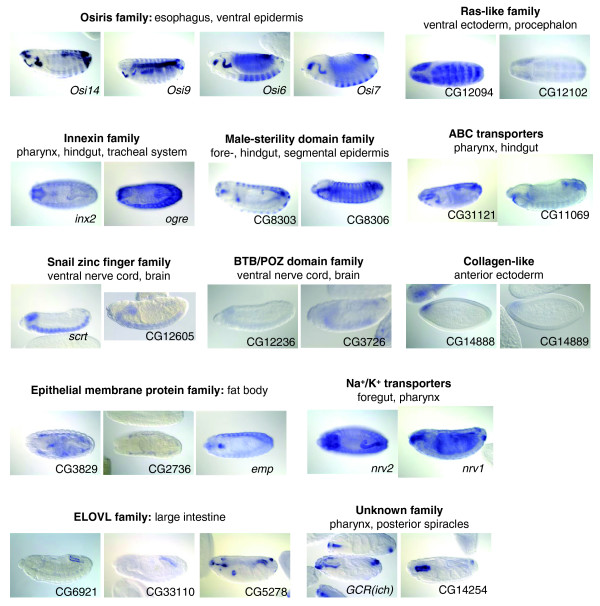
**Conserved TDGs show co-expression in the *Drosophila *embryo**. The figure shows *in situ *hybridization data for TDGs whose expression has not been previously described. Four genes from the *Osiris *cluster are expressed in the esophagus and in the ventral ectoderm, while three genes encoding Elongation-of-very-long-fatty-acids synthases (ELOVL) are expressed in the large intestine. We also found two undescribed genes that encode proteins with collagen-like repeats that are both expressed in a discrete domain at the anterior end of the syncitial blastoderm stage embryo, and two Ras-family members that show expression in the procephalon and ventral ectoderm. We have also found that the *scrt *Snail-type zinc finger gene has a conserved linked duplicate and both are expressed in overlapping domains in the central nervous system.

The set of evolutionarily conserved TDGs that are co-expressed includes many previously identified cases (Additional data file 11), such as *en *and *inv *[1], *tin *and *bap *[35], *gsb *and *gsb-n *[36], *srp *and *GATAe *[37], the *odd-drm-sob *zinc-finger cluster [38], and *wg *and *Wnt4 *[39]. However, a number of previously unreported cases of co-expression were also identified (Figure 3). Among these are four members of the *Osiris *gene family, which are expressed in common domains in the esophagus and the thoracic epidermis (Figure 3). These genes encode proteins of unknown function whose conservation with orthologues in *Anopheles *has been previously reported [40]. We also identified a pair of genes that encode previously undescribed proteins characterized by collagen-like triple helix repeats, and which are expressed at the early blastoderm stage in a highly restricted domain in the anterior pro-cephalic region (Figure 3). Another interesting example is that of the *Snail *family zinc-finger gene *scrt *and its duplicate pair CG12605 (Figure 3). Both genes are specifically expressed in the central nervous system, including the cephalic anlagen. *scrt *is considered to be a pan-neural marker in *Drosophila *development; however, its mutation produces only a subtle eye phenotype [41]. The fact that a closely related gene lies in its vicinity may indicate that the full function of *scrt *and CG12605 in neural development will not be revealed unless both genes are mutated (or deleted) simultaneously. Given the high number of tandem duplicates we have shown to be present in the *Drosophila *genome, this situation may be more frequent than previously thought, and might explain many cases of mutants that do not show the phenotype predicted on the basis of the wild-type gene's expression pattern.

## Conclusion

Our study provides evidence for the existence of evolutionary constraints that determine the relative positions of a large fraction of duplicated genes in the *Drosophila *genome; moreover our results show that this phenomenon is related to gene functionality. We have shown that duplicated pairs are extremely abundant, and that these pairs separate over evolutionary time according to an all-or-nothing pattern, which indicates that genome remodeling in *Drosophila *does not proceed by gradual separation. Despite the general trend for neutral gene shuffling, we found that duplicated pairs that are preferentially retained as neighbors are enriched in genes involved in developmental processes and the regulation of transcription. We further show that these conserved duplicated genes tend to be co-expressed in the early fly embryo, which suggests the existence of shared *cis*-acting regulatory regions that act as a selective brake to keep these gene copies in proximity.

This does not imply, however, that duplicated copies will remain fully redundant over time. It is not difficult to envision subsequent processes of neofunctionalization or subfunctionalization occurring between copies, which would result in clusters of two or more genes with partially overlapping expression domains and functions and at the same time specific and unique roles. This occurs with the Hox clusters, which are a clear example of conserved TDGs.

Nevertheless, it is unlikely that shared *cis*-regulation is the only mechanism acting to keep duplicates together, and it certainly does not exclude other constraints, such as chromatin structure, which may also play a role in the evolution of gene order.

Finally, the duplicated genes catalogued here will be of great value for the *Drosophila *community, since many of them may be involved in key developmental processes, and their characterization might help to uncover functions that are not apparent from simple forward genetics approaches, which overlook potential redundant roles of duplicated copies.

## Materials and methods

### Detection and classification of gene duplicates

We characterized the duplicate gene content of the *D. melanogaster *and *A. gambiae *genomes by following a series of simple steps. We first obtained the protein sets for each species from the April 2007 release of the Ensembl genome server [42] and, for those genes with multiple transcripts, filtered out shorter isoforms, retaining only the largest protein sequence for each gene. Second, we performed an intra-species comparison of all protein sequences using BLASTp [43], default options. In cases with several high scoring pairs (HSPs) per query, we obtained a single sum scored E-value for each matching protein pair by applying Karlin and Altschul statistics [44], which we also explain here. We have defined co-ordered HPSs by what we call 'best HSP tracking', which takes the best HSPs that are consistent with the coordinates of the immediately previous HSP, when they are ordered by E-value, starting with the most significant HSP. The E-value for one HSP is calculated with this statistic:

*E-value *= *kmne*^-*λS*^

where S is the score, m and n are the size of the query and the database size, respectively, lambda is a matrix specific constant to normalize the score and k is an adjusting constant of minor importance in the analysis. All the values can be captured from the BLAST output file. The sum score is calculated as:

$$S_{sum}=\lambda \sum_{i=1}^{r}S_{r}-\ln(kmn)-(r-1)(\ln(k)+2\ln(g)-\log(r!)$$

where m and n are the size of the query and the subject sequences, respectively, and g the gap size. The corresponding *p*-value then is:

$$p-value=e^{\left(-S_{sum}\right)}S_{sum}^{\left(r-1\right)}/(r!(r-1)!)$$

which can be corrected for multiple testing with:

*p-value*(*corr*) = *p-value*/*β*^(*r*-1) ^(1-*β*)

to finally obtain the corrected E-value (beta is the gap decay and 0.1 by default):

*E-value*(*corr*) = (*effective_db_lengtth*/*n*)*p-value*(*corr*)

Third, a gene pair was finally accepted as duplicated when their detected relation passed one of these two conditions: an E-value lower than 10^-20^; or the relation between identity and alignable sequence length passed the criteria described by Bukhard Rost [45] to exclude false positive relationships with no biological meaning (distance to Burkhard Rost > 0; see as follows). When the alignment length is lower than 300 amino acids the distance from a multiple HSP BLAST alignment to Burkhard Rost's homology estimation is:

*dist *= *percent_identity *- 10 - 480*alignment_length*^(-0.32(1+exp(-*alignment_length*/1000)))^

and for larger alignments we approximated the distance as:

*dist *= *percent_identity *- 30

TDGs were defined as duplicated genes separated by no more than ten non-related intervening genes, which provides a conservative value for significant linkage (Additional data file 2). Each chromosome arm was treated independently. An array of two or more consecutively linked genes meeting this criterion was defined as a TDG cluster or group. This step generated 1,001 groups in *Drosophila *and 899 in *Anopheles*, of which the vast majority are only composed of two or three genes (838 and 740, respectively, more than 80% in both cases; Additional data file 1).

To identify TDG clusters phylogenetically conserved between *Anopheles *and *Drosophila*, we first performed an all-versus-all BLASTp comparison of a joint collection of sequences containing all *Drosophila *and *Anopheles *proteins. We then ranked all the sequence relationships between each TDG cluster in *Drosophila *and each TDG cluster in *Anopheles *by their corresponding E-value. Conserved TDG clusters were defined as those for which more than 50% of all the possible interspecies relationships have lower E-values than intra-species hits. This filter ensured that practically all the *Drosophila *clusters identified as conserved have an ancient origin and thus avoided the inclusion of fly-specific duplicates. The conservation of TDG clusters with *D. pseudoobscura *and *D. virilis *was manually inspected by examining the genomic location of one-to-one orthologues with *D. melanogaster *on the University of California Santa Cruz (UCSC) Genome Browser [46]. For those TDG clusters of three or more genes, the presence of two genes in tandem was considered sufficient to be scored as conserved.

Because retrotransposed gene copies could potentially have an impact on our analysis and conclusions, we wanted to evaluate their relative abundance within our set of gene duplicates. A recently published work identifies only 94 retrogenes in the fly genome [16], a fraction that appears to be negligible if we consider all 8,664 duplicated genes used in this study. Nevertheless, to rule out without doubt that the contribution of retrogenes to our analysis is not relevant, we compared gene structures between duplicates in order to detect all possible episodes of retrotransposition within our set of duplicates. In total, we found 566 cases that could be compatible with a retrotranspositional origin (that is, that have no introns and a multiexonic paralog). To minimize the interference of possible insertions or deletions of introns in one of the copies with time, we repeated these comparisons by only considering 271 recent duplicates (dS < 0.1) and found that only approximately 10% of the cases (25 in total) are consistent with a retrotranspositional origin. This indicates that the contribution of retrotransposition in the appearance of gene duplicates in fly is marginal. Furthermore, and in agreement with this finding, we evaluated the distribution of the percentage of retrogenes (using this extremely relaxed definition) within our set using bins of 0.1 dS and observed that the percentage of retrogenes within our set of duplicates is always lower than 10% and normally between 5% and 6%. All the different sets of gene used in this study can be found in Additional data file 12.

### Calculation of dS values

We calculated the rate of synonymous substitutions (dS) between two particular gene copies by first extracting the alignment derived from the best-scoring HSPs obtained from their BLASTp comparison. Each of these alignments was then used as a template to obtain a codon-based DNA alignment (using their cDNA sequences and the pal2nal program [47]). Finally, dS values were calculated from the DNA alignments by maximum likelihood analysis using the *codeml *program included in the PAML package for phylogenetic analysis [48] (runmode = -2, seqtype = 1, and CodonFreq = F3 × 4).

In order to monitor potential issues derived from the saturation of high dS values, we also repeated all the analyses using dN values as a rough estimate of the relative age of duplicates. These dN values were extracted from the same alignments and the same PAML settings that yielded dS values.

The data for Figure 1b were fitted to an exponential one-phase association model using the GraphPad Prism^© ^package (La Jolla, California, USA), setting a Y0 value of 0 (to account for the fact that we are examining duplicates originated in tandem, and that by definition these will all be linked at time = 0). In these conditions, the 95% confidence interval for the plateau (asymptote) value was between 86.78% and 98.23%. When compared to a model with a hypothetical plateau value of 100%, the model where the distribution does not asymptote at 100% is statistically significant (*p*-value = 0.0242). If we use all of the available data for higher dS values (dS 0-7, or dS 0-10) we find that the distribution reaches a plateau at 89.89% of dispersed duplicates, with even higher significance compared to the hypothesis of asymptoting at 100% (*p*-value < 0.0001). Similar conclusions are reached if instead we divide the distribution in a two-phase linear model (not shown).

### Random test of gene ordering

To distinguish tandem from dispersed duplicates we needed to define the maximum distance (in i-genes) between two duplicated genes for which the linkage was significant when compared with a random distribution along the genome. For this, we modeled 10,000 random replicates of the *Drosophila *genome by shuffling all genes within the same chromosome arm while retaining their similarity values calculated as described above. We then calculated the probability of finding a particular separation in i-genes between two gene copies by chance by counting the frequency of such distances in all random models generated and dividing by the number of replicas (n = 10,000; Additional data file 2). To discard the influence of recently formed gene copies, which will mostly be in tandem (that is, at 0 i-genes) we also included, in addition to the test considering all duplicated genes, another test considering divergent gene copies only (taken here conservatively as copies with dS > 4).

### Gene Ontology analysis

The distributions of functions associated with the different subsets of gene duplicates were evaluated by analysis of GO terms [22] using the Fatigo tool from the Babelomics data analysis suite [49]. This tool provides an adjusted *p*-value based on family wise error rate and false discovery rate methods in order to correct for multiple tests [49]. Four high-level GO terms were selected for further examination. For a given subset of duplicate genes, the proportion of genes in each GO category was calculated by dividing the number of genes annotated with that category by the total number of genes in the subset. Values were normalized by dividing them by the proportion of genes annotated with that GO term in the complete set of duplicated genes (Additional data file 6). A value of 1 would indicate that the distribution of the GO term in question was the same in the given subset as in the complete set of duplicated genes. To compare the relative distributions of GO terms in the gene sets derived from Nelson *et al*. [28], values were normalized to the proportion of genes annotated with each GO term in the whole genome. This is because, by definition, the CDY set (single genes in *C. elegans*, *Drosophila *and yeast) is significantly underrepresented in the complete duplicated gene set.

### *In situ *analysis and scoring

For comparison of *in situ *hybridization staining patterns, duplicated genes were first categorized into the following subsets. (a) Conserved TDGs (see above). (b) Non-conserved TDGs. (c) Conserved non TDGs, defined as those dispersed duplicates in *Drosophila *(that is, separated by > 10 i-genes) for which each gene has a 1:1 orthologue in *Anopheles *(as defined in the Ensembl database). This subset includes duplicated genes that, like conserved TDGs, were formed before the separation of *Drosophila *and *Anopheles *lineages (and are thus of comparable ages); however, in this case, the duplicates have separated at least in the *Drosophila *lineage. (d) Conserved neighbors, which include genes not related by duplication that are located in regions of synteny between *D. melanogaster *and *A. gambiae *(obtained from the supplemental data to the honeybee genome analysis [27]) and that had 1:1 orthologues in *A. gambiae *(obtained as above). All TDGs were removed from this subset.

For each category, we identified those gene groups for which at least two genes had been tested for embryonic expression by whole mount *in situ *hybridization (Table 1). The expression data for all genes in a given group were visually inspected and compared. A gene group was scored as positive if at least two genes showed expression in a common domain or sub-domain of the embryo. Other groups were scored as not informative; we deliberately did not search for negative evidence, since we cannot exclude the possibility that two genes that are not expressed in common domains in the embryo stages examined do so at other unexplored stages or in the adult. Therefore, we can only score for positive evidence of co-expression. Maternal or ubiquitous expression was not used as positive evidence for co-expression.

## Abbreviations

dN: number of non-synonymous substitutions per site; dS: number of synonymous substitutions per site; GO: Gene Ontology; HSP: high-scoring pair; TDG: tandemly arrayed duplicated gene.

## Authors' contributions

DT, MMil and MMan conceived the study. CQ, DT and PB designed the computational analysis. CQ, JL-M and MS carried out the computational analysis. PT, MMil and MMan analyzed the *Drosophila *expression data. CQ, DT and MMan drafted the manuscript. All authors read and approved the final manuscript.

## Additional data files

The following additional data are available with the online version of this paper. Additional data file 1 is a figure showing the distribution of paralogous gene group size and distance. Additional data file 2 is a figure showing the statistical test used to define tandemly and dispersed duplicated genes. Additional data file 3 is a table listing over- and underrepresented GO categories in TDGs subdivided by dS ranges, compared to dispersed duplicates. Additional data file 4 is a table listing over- and underrepresented GO categories in TDGs compared to dispersed duplicates. Additional data file 5 is a table listing over- and underrepresented GO categories in conserved TDGs compared to dispersed duplicates. Additional data file 6 is a table listing the number of *D. melanogaster *genes included in each category of selected GO terms and gene sets. Additional data file 7 is a figure showing that evolutionarily conserved TDGs are enriched in 'complex' genes. Additional data file 8 is a table listing the groups of TDGs conserved between *D. melanogaster *and *A. gambia*e used in the embryonic co-expression analysis. Additional data file 9 is a table listing the groups of genes in conserved linkage between *D. melanogaster *and *A. gambia*e that are not tandem duplicates used in the embryonic co-expression analysis. Additional data file 10 is a short discussion on the stringency in the definition of conserved TDGs as used in this study. Additional data file 11 is a figure showing the conserved TDGs that are co-expressed in the *Drosophila *embryo that have been previously described in the literature. Additional data file 12 is a compressed file containing the different *D. melanogaster *gene sets used in this study as plain text.

## Supplementary Material

Additional data file 1 Distribution of paralogous gene group size and distance. Click here for file

Additional data file 2 Statistical test used to define tandemly and dispersed duplicated genes. Click here for file

Additional data file 3 Over- and underrepresented GO categories in TDGs subdivided by dS ranges, compared to dispersed duplicates. Click here for file

Additional data file 4 Over- and underrepresented GO categories in TDGs compared to dispersed duplicates. Click here for file

Additional data file 5 Over- and underrepresented GO categories in conserved TDGs compared to dispersed duplicates. Click here for file

Additional data file 6 Number of *D. melanogaster *genes included in each category of selected GO terms and gene sets. Click here for file

Additional data file 7 Evolutionarily conserved TDGs are enriched in 'complex' genes. Click here for file

Additional data file 8 Groups of TDGs conserved between *D. melanogaster *and *A. gambia*e used in the embryonic co-expression analysis. Click here for file

Additional data file 9 Groups of genes in conserved linkage between *D. melanogaster *and *A. gambia*e that are not tandem duplicates used in the embryonic co-expression analysis. Click here for file

Additional data file 10 Stringency in the definition of conserved TDGs as used in this study. Click here for file

Additional data file 11 Conserved TDGs that are co-expressed in the *Drosophila *embryo that have been previously described in the literature. Click here for file

Additional data file 12 *D. melanogaster *gene sets used in this study. Click here for file
