# Supplementary material for: Selective maintenance of Drosophila tandemly arranged duplicated genes during evolution
Source: Genome Biol. 2008 Dec 16;9(12):R176. doi: 10.1186/gb-2008-9-12-r176 (PMC2646280; doi:10.1186/gb-2008-9-12-r176)
Supplement: Additional data file 1 — Distribution of paralogous gene group size and distance. [file gb-2008-9-12-r176-S1.pdf]

a

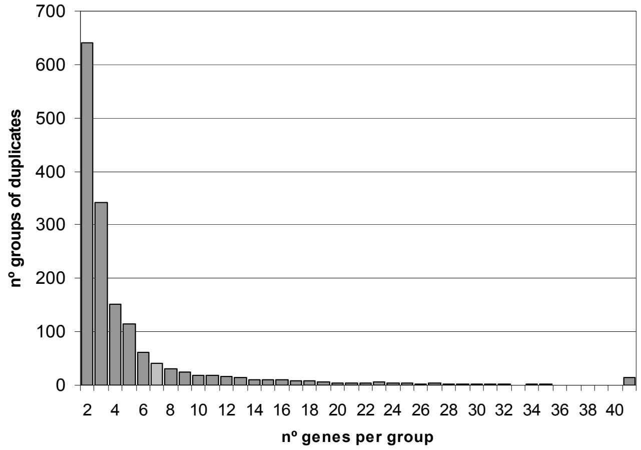

b

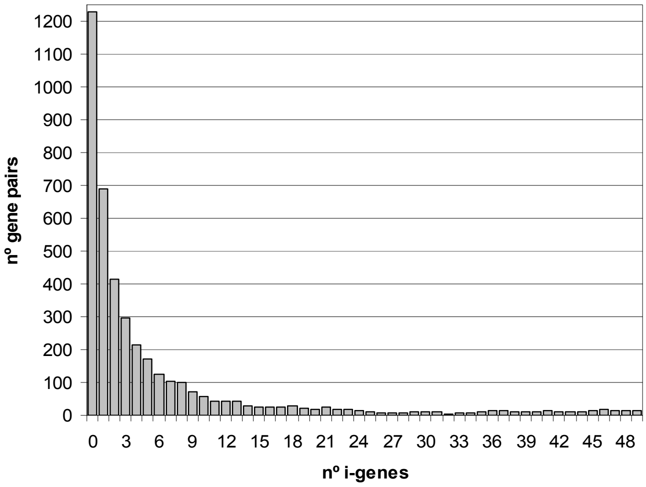

Additional Figure 1. Distribution of paralogous gene group size and distance. (**a**) The estimated number of groups for each possible group size shows an L-shaped distribution, similar to what is obtained from hierarchical clustering of genes into isolated families. Our analysis takes into consideration that a gene can belong to two or more different groups based on sequence similarity. The last data point represents groups with more than 41 members. (**b**) There are many gene pairs located at a short distance (measured by the number of intervening non paralogous genes separating two paralogous genes) and an exponential decrease in the number of pairs located at larger distances. Note that the graph only includes pairs separated by up to 50 intervening genes, and that overall the majority of gene pairs are not physically linked.
