# Supplementary material for: Selective maintenance of Drosophila tandemly arranged duplicated genes during evolution
Source: Genome Biol. 2008 Dec 16;9(12):R176. doi: 10.1186/gb-2008-9-12-r176 (PMC2646280; doi:10.1186/gb-2008-9-12-r176)
Supplement: Additional data file 2 — Statistical test used to define tandemly and dispersed duplicated genes. [file gb-2008-9-12-r176-S2.pdf]

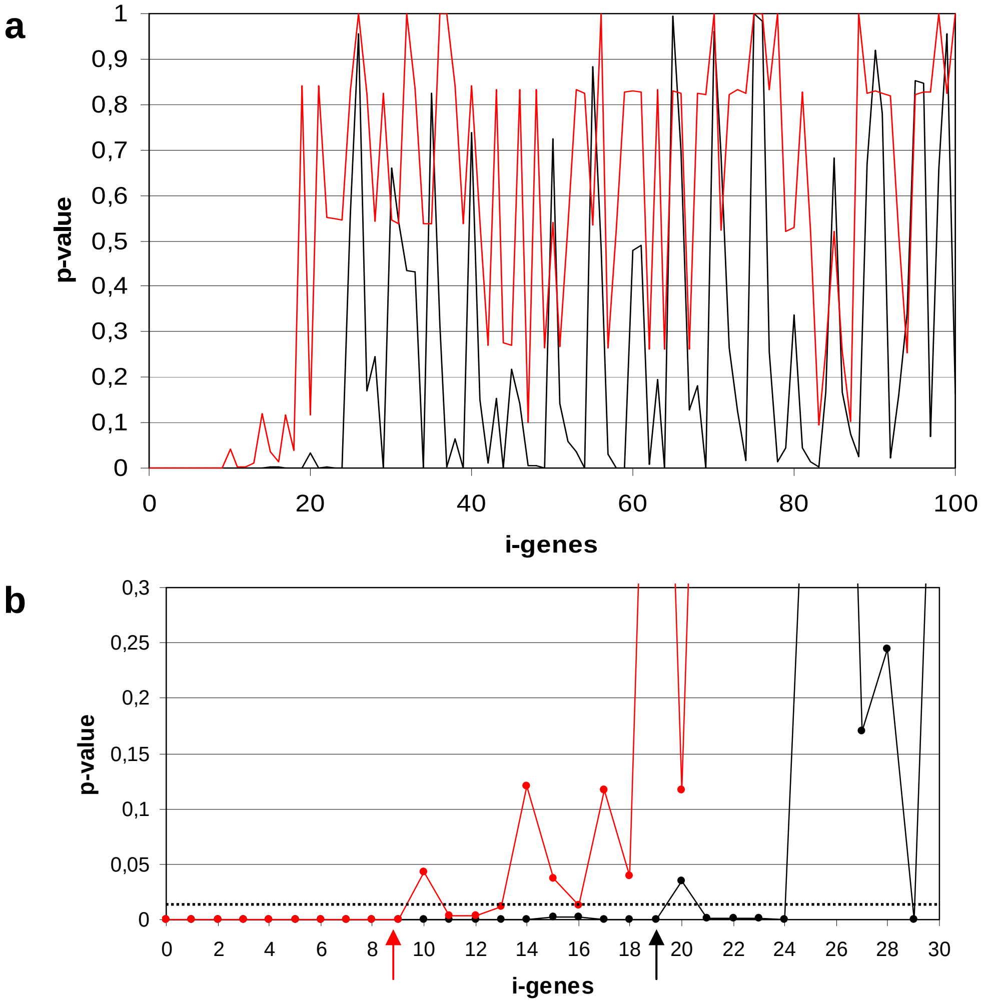

Additional Figure 2. Statistical test to define tandemly and dispersed duplicated genes. (**a**, **b**) The probability to find the same distribution of TDGs present in the D. melanogaster genome in 10,000 randomly reshuffled genomes, plotted against distance in the same chromosome in intervening genes (i-genes). There is high statistical support (p<0,01) to define as tandems those genes that are separated by up to 19 intervening genes (black line). This value is of 9 intervening genes when only the most divergent (dS > 4) relations are used (red line). (**b**) is an enlargement of (**a**) for values of 0 to 30 intervening genes and p-values of 0 to 0.3. The dotted line indicates a p-value of 0,01.
