# Supplementary material for: Selective maintenance of Drosophila tandemly arranged duplicated genes during evolution
Source: Genome Biol. 2008 Dec 16;9(12):R176. doi: 10.1186/gb-2008-9-12-r176 (PMC2646280; doi:10.1186/gb-2008-9-12-r176)
Supplement: Additional data file 3 — Over- and underrepresented GO categories in TDGs subdivided by dS ranges, compared to dispersed duplicates. [file gb-2008-9-12-r176-S3.pdf]

Additional Table 1

Over and under represented GO categories in TDGs subdivided by dS ranges, compared to dispersed duplicates

Over represented GO terms 0<dS<2	Level	adj. p-value
chromatin assembly	9	3.689584e-79
protein-DNA complex assembly	5	3.228133e-71
DNA binding	4	3.555438e-23
trypsin activity	7	9.218969e-08
protein kinase CK2 regulator activity	5	9.218969e-08
chymotrypsin activity	7	5.085664e-07
phospholipase A1 activity	8	2.726116e-06
meiotic chromosome segregation	9	1.871472e-05
cytolysis	7	3.187966e-05
proteolysis	6	4.004771e-05
defense response to Gram-negative bacterium	7	1.009030e-04
glutathione transferase activity	5	2.133344e-04
innate immune response	5	2.385094e-04
female meiosis	9	2.861814e-04
heme binding	4	4.815625e-04
antibacterial humoral response (sensu Protostomia)	8	9.186038e-04
iron ion binding	6	1.527452e-03
nervous system development	5	3.288158e-03
membrane alanyl aminopeptidase activity	8	7.316578e-03
lysozyme activity	6	9.700135e-03
neurotransmitter:sodium symporter activity	9	1.590215e-02
structural constituent of larval cuticle (sensu Insecta)	5	3.029712e-02
monooxygenase activity	4	3.501156e-02
ion channel inhibitor activity	5	4.498029e-02
cell wall catabolic process	5	4.934063e-02

Under represented GO terms 0<dS<2	Level	adj. p-value
protein binding	3	3.088699e-15
RNA binding	4	1.580750e-05
ubiquitin cycle	7	5.055034e-05
cell morphogenesis	5	2.217040e-04
negative regulation of cellular process	5	2.183296e-03
RNA processing	6	2.205736e-03
cellular lipid metabolic process	5	2.381012e-03
pyrophosphatase activity	6	2.961933e-03
oogenesis (sensu Insecta)	7	2.974796e-03
cation channel activity	6	3.503477e-03
wing morphogenesis	9	3.616954e-03
transferase activity, transferring phosphorus-containing groups	4	3.916381e-03
macromolecule biosynthetic process	5	4.222194e-03
cell division	3	4.440102e-03
regulation of developmental process	4	9.382316e-03
regulation of signal transduction	5	1.095682e-02
cell adhesion	3	1.205008e-02
embryonic development	4	1.505409e-02
axis specification	5	1.898928e-02
germ cell development	6	2.305569e-02
muscle development	6	2.305569e-02
establishment of cellular localization	5	2.389140e-02
phosphorylation	6	2.451898e-02

eye morphogenesis (sensu Endopterygota)	9	2.561319e-02
adenyl nucleotide binding	5	3.029712e-02
small conjugating protein ligase activity	6	3.204147e-02
actin cytoskeleton organization and biogenesis	7	3.508915e-02
phosphoric monoester hydrolase activity	6	3.511294e-02
protein amino acid dephosphorylation	7	3.740092e-02
GTPase regulator activity	3	3.943621e-02
synaptic transmission	6	3.995742e-02
potassium ion transport	8	4.774955e-02
biopolymer catabolic process	5	4.774955e-02
synaptic vesicle transport	6	4.781702e-02

Over represented GO terms 2<dS<4	Level	adj. p-value
proteolysis	6	5.570009e-31
trypsin activity	7	1.502997e-13
glutathione transferase activity	5	3.630118e-10
oxidoreductase activity, acting on paired donors…	5	2.968221e-05
chymotrypsin activity	7	4.748059e-05
lipid catabolic process	5	1.446508e-04
chitin metabolic process	9	1.953686e-04
alpha-amylase activity	7	5.660935e-04
phospholipase A1 activity	8	8.837874e-04
cytolysis	7	1.104212e-03
defense response to Gram-negative bacterium	7	1.118183e-03
antibacterial humoral response (sensu Protostomia)	8	1.186524e-03
heme binding	4	2.035134e-03
pattern binding	3	2.060777e-03
aminoacylase activity	6	3.802387e-03
peptidoglycan catabolic process	7	3.859496e-03
larval cuticle biosynthetic process (sensu Insecta)	9	3.859496e-03
carboxypeptidase A activity	8	5.105679e-03
mannose metabolic process	8	7.282256e-03
peptide receptor activity, G-protein coupled	7	1.859617e-02
innate immune response	5	1.960458e-02
elastase activity	6	2.148667e-02
monooxygenase activity	4	2.222876e-02
G-protein coupled receptor protein signaling pathway	6	2.325188e-02
structural constituent of larval cuticle (sensu Insecta)	5	3.232174e-02
alpha-mannosidase activity	7	3.355407e-02
N-acetylmuramoyl-L-alanine amidase activity	6	3.707965e-02
lysozyme activity	6	4.417100e-02
neurotransmitter:sodium symporter activity	9	4.977773e-02

Under represented GO terms 2<dS<4	Level	adj. p-value
ATP binding	6	8.591315e-17
protein modification	6	4.251236e-06
DNA metabolic process	5	3.544747e-05
regulation of cellular process	4	5.156865e-05
RNA binding	4	3.072598e-04
cytoskeleton organization and biogenesis	5	1.493384e-03
helicase activity	3	6.722672e-03
phosphotransferase activity, alcohol group as acceptor	5	6.722672e-03
macromolecule complex assembly	4	7.282256e-03

phosphate metabolic process	5	7.658703e-03
synaptic transmission	6	1.395499e-02
chromosome organization and biogenesis (sensu Eukaryota)	6	1.395499e-02
metal ion binding	4	1.585043e-02
DNA binding	4	1.769592e-02
regulation of metabolic process	4	1.960458e-02
protein transport	5	2.298740e-02
secretion	4	2.619186e-02
guanyl nucleotide binding	5	2.847739e-02
M phase	6	2.862528e-02
ATPase activity	8	3.025879e-02
RNA metabolic process	5	3.305701e-02
intracellular transport	6	3.405999e-02
oogenesis	6	3.405999e-02
ion transport	5	3.548623e-02
ubiquitin-protein ligase activity	7	3.580240e-02
cation binding	4	3.671019e-02
cytoskeletal protein binding	4	3.752242e-02
cell morphogenesis	5	3.773052e-02
system development	4	4.839224e-02
cysteine-type peptidase activity	5	4.994030e-02
unfolded protein binding	4	4.994030e-02

Over represented GO terms<br>dS>4	Level	adj. p-value
proteolysis	6	2.019770e-41
trypsin activity	7	1.344451e-21
structural constituent of cuticle	3	1.099307e-17
chitin metabolic process	9	2.410592e-12
oxidoreductase activity, acting on paired donors…	5	1.713796e-06
glutathione transferase activity	5	1.713796e-06
chymotrypsin activity	7	1.581338e-05
chitin binding	5	1.603990e-05
alpha-amylase activity	7	1.338765e-04
regulation of transcription, DNA-dependent	8	9.491890e-04
protein amino acid phosphorylation	7	9.776096e-04
lipid catabolic process	5	1.161056e-03
cytolysis	7	2.740274e-03
sensory perception of taste	6	4.923385e-03
leg joint morphogenesis	9	1.066244e-02
mesoderm formation	9	1.120510e-02
leg disc morphogenesis	8	1.546698e-02
fatty acid biosynthetic process	8	2.120323e-02
defense response	3	2.417198e-02
periodic partitioning by pair rule gene	9	2.488814e-02
lymph gland development (sensu Arthropoda)	8	2.728903e-02
embryonic hemopoiesis	9	3.171408e-02
cellular polysaccharide catabolic process	7	3.554490e-02
N-acetylglucosamine catabolic process	9	3.631879e-02
head segmentation	8	3.717381e-02
segment specification	7	4.307098e-02

Under represented GO terms<br>dS>4	Level	adj. p-value
GTP binding	6	4.983906e-13

RNA binding	4	6.296507e-08
small GTPase mediated signal transduction	6	3.346444e-06
ubiquitin-protein ligase activity	7	5.051943e-05
nucleoside-triphosphatase activity	7	5.796976e-05
helicase activity	3	6.639776e-05
vesicle-mediated transport	5	1.007566e-04
ATP binding	6	1.030653e-04
phosphoprotein phosphatase activity	7	1.499030e-04
calcium ion binding	5	4.620100e-04
nucleotidyltransferase activity	5	5.729702e-04
RNA processing	6	7.380614e-04
DNA replication	6	8.628258e-04
M phase	6	8.628258e-04
cell division	3	1.105000e-03
acyltransferase activity	6	1.396066e-03
protein folding	6	1.742417e-03
ubiquitin cycle	7	1.784334e-03
unfolded protein binding	4	1.869192e-03
structural constituent of ribosome	3	3.313543e-03
peptidyl-prolyl cis-trans isomerase activity	5	3.765359e-03
meiotic cell cycle	4	4.282376e-03
translation factor activity, nucleic acid binding	4	5.057468e-03
intracellular protein transport	7	6.115502e-03
DNA repair	6	7.237108e-03
mitotic cell cycle	4	7.237108e-03
cell morphogenesis	5	7.348334e-03
aminoacyl-tRNA ligase activity	6	7.506077e-03
dephosphorylation	6	1.032527e-02
cysteine-type endopeptidase activity	6	1.081741e-02
chromosome organization and biogenesis (sensu Eukaryota)	6	1.120510e-02
ribonucleoprotein complex biogenesis and assembly	4	1.355452e-02
enzyme activator activity	3	1.777950e-02
guanyl-nucleotide exchange factor activity	4	1.777950e-02
voltage-gated ion channel activity	6	1.891972e-02
cation transporter activity	4	2.020619e-02
tube morphogenesis	5	2.033391e-02
microtubule-based process	6	2.120323e-02
response to abiotic stimulus	3	2.488814e-02
gametogenesis	4	2.613665e-02
mRNA metabolic process	6	2.649943e-02
DNA packaging	6	2.728903e-02
heterocycle metabolic process	4	2.822923e-02
neurotransmitter secretion	8	2.838866e-02
protein transporter activity	3	3.088443e-02
cofactor metabolic process	4	3.283787e-02
tRNA aminoacylation for protein translation	9	3.446885e-02
protein complex assembly	5	3.666042e-02
membrane invagination	5	3.666042e-02
methyltransferase activity	5	4.065713e-02
enzyme binding	4	4.081799e-02
actin binding	5	4.663397e-02
regulation of progression through cell cycle	6	4.751564e-02

Only “Biological Processes” and “Molecular Function” categories were used.
Only non-redundant terms with an adjusted p-value <0.05 are shown.
GO terms related to development and differentiation are highlighted in gray, and those related to transcriptional regulation in blue.
