# Supplementary material for: Selective maintenance of Drosophila tandemly arranged duplicated genes during evolution
Source: Genome Biol. 2008 Dec 16;9(12):R176. doi: 10.1186/gb-2008-9-12-r176 (PMC2646280; doi:10.1186/gb-2008-9-12-r176)
Supplement: Additional data file 4 — Over- and underrepresented GO categories in TDGs compared to dispersed duplicates. [file gb-2008-9-12-r176-S4.pdf]

Additional Table 2

Over and under represented GO categories in TDGs compared to dispersed duplicates

Over represented GO terms	Level	adj. p-value
chromatin assembly	9	2.221977e-33
protein-DNA complex assembly	5	1.508135e-28
trypsin activity	7	1.139258e-19
structural constituent of cuticle	3	3.313147e-18
chitin metabolic process	9	5.689048e-09
chitin binding	5	1.622031e-06
oxidoreductase activity, acting on paired donors	5	3.697661e-06
chymotrypsin activity	7	4.921593e-06
glutathione transferase activity	5	8.525285e-06
alpha-amylase activity	7	7.383382e-05
DNA binding	4	1.000654e-04
heme binding	4	3.001551e-04
membrane alanyl aminopeptidase activity	8	3.377677e-04
protein amino acid dephosphorylation	7	3.381429e-04
ATP binding	6	3.839177e-04
protein serine/threonine kinase activity	7	1.297425e-03
phospholipase A1 activity	8	1.642339e-03
protein amino acid phosphorylation	7	1.838134e-03
cytolysis	7	2.401718e-03
antibacterial humoral response (sensu Protostomia)	8	3.830717e-03
carboxypeptidase A activity	8	8.212148e-03
serine-type endopeptidase inhibitor activity	6	8.417196e-03
taste receptor activity	7	9.012502e-03
iron ion binding	6	1.033587e-02
elastase activity	6	1.177254e-02
protein kinase CK2 regulator activity	5	1.435802e-02
defense response to Gram-negative bacterium	7	1.508369e-02
lipid catabolic process	5	3.511967e-02
innate immune response	5	3.511967e-02
blastoderm segmentation	7	3.762113e-02
sensory perception of taste	6	3.951579e-02
embryonic heart tube development	7	4.459342e-02

Under represented GO terms	Level	adj. p-value
ubiquitin cycle	7	6.688563e-07
helicase activity	3	6.069607e-06
nucleoside-triphosphatase activity	7	2.906415e-05
phosphoprotein phosphatase activity	7	3.185056e-05
GTP binding	6	3.737752e-05
ubiquitin-protein ligase activity	7	3.790028e-05
DNA replication	6	8.141656e-05
unfolded protein binding	4	3.001551e-04
peptidyl-prolyl cis-trans isomerase activity	5	3.927342e-04
cytoskeletal protein binding	4	5.022461e-04
tRNA aminoacylation for protein translation	9	6.232828e-04
aminoacyl-tRNA ligase activity	6	6.328441e-04
guanyl-nucleotide exchange factor activity	4	8.398182e-04
cation transport	6	1.291837e-03
protein folding	6	1.359748e-03
small GTPase mediated signal transduction	6	1.539161e-03
voltage-gated ion channel activity	6	1.741152e-03

cysteine-type endopeptidase activity	6	2.126571e-03
nucleotidyltransferase activity	5	2.207494e-03
neurotransmitter secretion	8	2.707491e-03
oogenesis	6	3.093372e-03
enzyme binding	4	3.562912e-03
intracellular protein transport	7	4.553116e-03
cell morphogenesis	5	4.734493e-03
acyltransferase activity	6	6.405960e-03
GTPase activator activity	4	7.948672e-03
oxidoreductase activity, acting on NADH or NADPH	4	7.948672e-03
regulation of progression through cell cycle	6	1.143647e-02
calcium ion binding	5	1.177254e-02
regulation of protein metabolic process	5	1.261579e-02
synaptic vesicle endocytosis	7	1.366790e-02
methyltransferase activity	5	1.568451e-02
mitotic cell cycle	4	1.794113e-02
DNA repair	6	1.813100e-02
electron carrier activity	4	1.995588e-02
microtubule-based process	6	2.590362e-02
regulation of signal transduction	5	2.961335e-02
regulation of gene expression, epigenetic	4	3.350320e-02
heterocycle metabolic process	4	3.350320e-02
actin cytoskeleton organization and biogenesis	7	3.411413e-02
smoothened signaling pathway	6	3.411413e-02
regulation of catalytic activity	4	3.430634e-02
response to radiation	4	3.430634e-02
tube morphogenesis	5	3.511967e-02
negative regulation of biological process	4	3.511967e-02
translation elongation factor activity	5	3.615328e-02
cofactor metabolic process	4	3.939710e-02
dipeptidyl-peptidase and tripeptidyl-peptidase activity	6	4.145648e-02
potassium channel activity	7	4.175374e-02
exonuclease activity	6	4.312886e-02
modification-dependent protein catabolic process	9	4.326510e-02
mRNA processing	7	4.326510e-02
mRNA binding	5	4.541545e-02
cytokinesis	4	4.607470e-02
heat shock protein binding	4	4.965611e-02
protein-RNA complex assembly	5	4.968409e-02
vesicle organization and biogenesis	5	4.968409e-02
protein polymerization	6	4.968409e-02

Only “Biological Processes” and “Molecular Function” categories were used.

Only non-redundant terms with an adjusted p-value <0.05 are shown.

GO terms related to development and differentiation are highlighted in gray, and those related to transcriptional regulation in blue.
