# Supplementary material for: Selective maintenance of Drosophila tandemly arranged duplicated genes during evolution
Source: Genome Biol. 2008 Dec 16;9(12):R176. doi: 10.1186/gb-2008-9-12-r176 (PMC2646280; doi:10.1186/gb-2008-9-12-r176)
Supplement: Additional data file 5 — Over- and underrepresented GO categories in conserved TDGs compared to dispersed duplicates. [file gb-2008-9-12-r176-S5.pdf]

Additional Table 3

Over and under represented GO categories in conserved TDGs compared to dispersed duplicates

Over represented GO terms	Level	adj. p-value
protein amino acid phosphorylation	7	6.357202e-15
trypsin activity	7	1.454860e-11
protein serine/threonine kinase activity	7	1.454860e-11
structural constituent of cuticle	3	1.352095e-05
cell fate specification	6	9.342389e-05
sequence-specific DNA binding	5	1.401508e-04
mesoderm formation	9	1.682214e-04
leg joint morphogenesis	9	3.006959e-04
transcription factor activity	5	5.054698e-04
midgut development	7	1.024239e-03
regulation of transcription from RNA polymerase II promoter	9	1.063909e-03
positive regulation of transcription, DNA-dependent	9	2.450942e-03
leg morphogenesis (sensu Endopterygota)	9	3.172980e-03
transcriptional activator activity	3	3.432137e-03
cardioblast differentiation	8	4.088958e-03
lymph gland development (sensu Arthropoda)	8	4.088958e-03
periodic partitioning by pair rule gene	9	5.118436e-03
hemocyte differentiation (sensu Arthropoda)	9	5.180785e-03
proteolysis	6	5.472519e-03
gonadal mesoderm development	8	1.185432e-02
head segmentation	8	1.185432e-02
embryonic heart tube development	7	1.287970e-02
cuticle biosynthetic process	5	1.294956e-02
embryonic hemopoiesis	9	1.419741e-02
regulation of cell differentiation	5	2.370797e-02
genital disc development	7	2.434739e-02
frizzled-2 signaling pathway	7	2.434739e-02
ATP binding	6	3.500974e-02
neurotransmitter receptor activity	4	4.061725e-02
specification of segmental identity, trunk	9	4.070931e-02

Under represented GO terms	Level	adj. p-value
cellular biosynthetic process	4	8.787626e-04
generation of precursor metabolites and energy	4	1.941079e-02
phosphoric monoester hydrolase activity	6	3.325736e-02
protein transport	5	3.672855e-02
cation transport	6	4.060544e-02
RNA binding	4	4.061725e-02
protein folding	6	4.905897e-02

Over and under represented GO categories in conserved TDGs compared to non-conserved TDGs

Over represented GO terms	Level	adj. p-value
protein amino acid phosphorylation	7	9.245947e-13
ATP binding	6	6.788585e-11
protein serine/threonine kinase activity	7	1.635591e-09
positive regulation of transcription, DNA-dependent	9	7.634051e-05
mesoderm formation	9	1.028943e-04
sequence-specific DNA binding	5	1.114247e-04
regulation of transcription from RNA polymerase II promoter	9	1.429476e-04
regulation of cell shape	7	2.252113e-04
cell fate specification	6	3.739495e-04
midgut development	7	8.702165e-04
embryonic morphogenesis	5	1.072847e-03
transcription factor activity	5	1.233735e-03
negative regulation of cellular process	5	1.281967e-03
leg morphogenesis (sensu Endopterygota)	9	2.114021e-03
leg joint morphogenesis	9	2.119958e-03
neurotransmitter receptor activity	4	5.073172e-03
oogenesis	6	5.081046e-03
pyrophosphatase activity	6	5.166931e-03
transcriptional activator activity	3	5.308557e-03
cardioblast differentiation	8	6.081960e-03
zinc ion binding	6	8.276799e-03
positive regulation of programmed cell death	9	9.738135e-03
gonadal mesoderm development	8	9.738135e-03
muscle development	6	9.780152e-03
axis specification	5	1.036244e-02
embryonic organ development	6	1.037107e-02
regulation of apoptosis	9	1.280132e-02
frizzled-2 signaling pathway	7	1.311470e-02
carboxylic acid transporter activity	4	1.452508e-02
central nervous system development	6	1.678360e-02
cell proliferation	3	1.827000e-02
periodic partitioning by pair rule gene	9	2.047112e-02
lymph gland development (sensu Arthropoda)	8	2.068215e-02
regulation of cell differentiation	5	2.218000e-02
hemocyte differentiation (sensu Arthropoda)	9	2.420941e-02
acid-amino acid ligase activity	5	2.483924e-02
hydrolase activity, hydrolyzing O-glycosyl compounds	5	2.739716e-02
cuticle biosynthetic process	5	3.000189e-02
actin cytoskeleton organization and biogenesis	7	3.025900e-02
segment specification	7	3.025900e-02
amino acid transport	7	3.025900e-02
segment polarity determination	9	3.068473e-02
germ cell migration	6	3.343154e-02
chromosome condensation	6	3.475204e-02
transcriptional repressor activity	3	3.637231e-02
positive regulation of cell growth	9	3.972741e-02
wing disc anterior/posterior pattern formation	9	3.972741e-02
pericardial cell differentiation	8	3.972741e-02
genital disc development	7	4.234800e-02

Under represented GO terms	Level	adj. p-value
chromatin assembly	9	9.972117e-10
protein-DNA complex assembly	5	9.106931e-08
iron ion binding	6	9.797565e-04
oxidoreductase activity	3	1.233735e-03
sensory perception of chemical stimulus	5	4.909961e-03
chitin metabolic process	9	1.280132e-02
electron transport	5	2.477310e-02
metalloexopeptidase activity	6	3.434394e-02
transferase activity, transferring hexosyl groups	5	3.747480e-02
cellular catabolic process	4	4.182430e-02
carbohydrate binding	3	4.335095e-02

Only "Biological Processes" and "Molecular Function" categories were used.

Only non-redundant terms with an adjusted p-value <0.05 are shown.

GO terms related to development and differentiation are highlighted in gray, and those related to transcriptional regulation in blue.
