# Supplementary material for: Selective maintenance of Drosophila tandemly arranged duplicated genes during evolution
Source: Genome Biol. 2008 Dec 16;9(12):R176. doi: 10.1186/gb-2008-9-12-r176 (PMC2646280; doi:10.1186/gb-2008-9-12-r176)
Supplement: Additional data file 6 — Number of D. melanogaster genes included in each category of selected GO terms and gene sets. [file gb-2008-9-12-r176-S6.pdf]

## Additional Table 4

**Number of *D. melanogaster* genes included in each category of selected GO terms and gene sets**

| | **All** | **Catalytic activity GO:0003824** | **Metabolic process GO:0008152** | **Mult. org. developmental GO:0007275** | **Transcription reg. activity GO:0030528** | **CDY set[a]** | **HK set[a]** | **Complex set[a]** |
|---|---|---|---|---|---|---|---|---|
| **All** | 14703 | 3292 | 4231 | 1271 | 421 | 1233 | 601 | 257 |
| **Duplicates** | 8664 | 2686 | 3230 | 993 | 337 | 653 | 401 | 234 |
| **TDGs** | 2952 | 953 | 1132 | 256 | 112 | 54 | 42 | 56 |
| **non TDGs** | 4784 | 1436 | 1723 | 569 | 180 | 529 | 307 | 117 |
| **conserved TDGs** | 400 | 125 | 152 | 86 | 40 | 18 | 8 | 21 |
| **non conserved TDGs** | 2552 | 828 | 980 | 170 | 72 | 36 | 34 | 35 |

[a] Data obtained from Nelson et al. (2004).
