# Supplementary material for: Selective maintenance of Drosophila tandemly arranged duplicated genes during evolution
Source: Genome Biol. 2008 Dec 16;9(12):R176. doi: 10.1186/gb-2008-9-12-r176 (PMC2646280; doi:10.1186/gb-2008-9-12-r176)
Supplement: Additional data file 7 — Evolutionarily conserved TDGs are enriched in 'complex' genes. [file gb-2008-9-12-r176-S7.pdf]

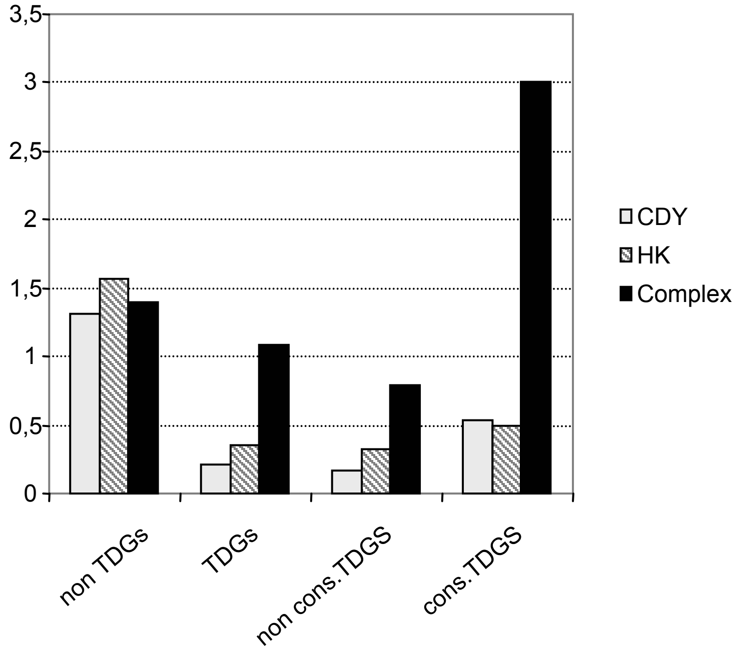

Additional Figure 3. Evolutionarily conserved TDGs are enriched in "complex" genes while housekeeping (HK) and CDY genes are underrepresented. Shown is the ratio of each of the categories defined by Nelson et al (2004) in the different subsets of duplicated genes relative, compared to all genes. A value of 1 indicates that the abundance in a subset is comparable to that in the whole genome. CDY set, light grey bar; HK set, stippled bar; Complex set, black bar. non TDGs, duplicated genes that are not arranged in tandem: TDGs, duplicated genes that are arranged in tandem; non cons. TDGs, tandem duplicates that are not conserved in *A. gambiae*; cons. TDGs, tandem duplicates that are conserved between *D. melanogaster* and *A. gambiae*.
