# Supplementary material for: Selective maintenance of Drosophila tandemly arranged duplicated genes during evolution
Source: Genome Biol. 2008 Dec 16;9(12):R176. doi: 10.1186/gb-2008-9-12-r176 (PMC2646280; doi:10.1186/gb-2008-9-12-r176)
Supplement: Additional data file 8 — Groups of TDGs conserved between D. melanogaster and A. gambiae used in the embryonic co-expression analysis. [file gb-2008-9-12-r176-S8.pdf]

Additional Table 5

Groups of TDGs conserved between *D. melanogaster* and *A. gambiae* used in the embryonic co-expression analysis

1. Groups showing co-expression

nº co-expressed genes /nº genes in group*	site of co-expression	*genes in group for which in situ hybridization in the early embryo has been performed
2/2	anterior ectoderm	CG14888 CG14889
2/2	posterior segments	CG10388 CG10325
2/2	segment-polarity	CG9015 CG17835
2/2	segment-polarity	CG3388 CG2692
2/2	pharynx, hindgut	CG31121 CG11069
2/2	anterior and posterior endoderm	CG3992 CG10278
2/2	ventral nerve cord and brain	CG1130 CG12605
2/2	muscle precursors	CG7895 CG7902
2/2	ventral ectoderm and procephalon	CG12094 CG12102
2/2	ventral nerve cord and brain	CG12236 CG3726
2/2	pharynx, posterior spiracles	CG5812 CG14254
2/2	segment-polarity	CG4889 CG4698
2/2	fore- and hindgut, epidermis	CG8303 CG8306
2/2	foregut and pharynx	CG9261 CG9258
2/3	pharynx, hindgut and tracheal system	CG4590 CG3039 CG2977
3/3	segment-polarity	CG3851 CG10016 CG3242
3/3	fat body	CG3829 CG2727 CG2736
3/4	large intestine	CG6921 CG33110 CG5278 CG5326
4/5	esophagus, ventral epidermis	CG1155 CG1154 CG15592 CG1151 CG1153

2. Groups with no evidence for co-expression

**Genes in group for which in situ hybridization in the early embryo has been performed**
CG31522 CG31523
CG7675 CG31235
CG18754 CG16705
CG16876 CG33119
CG5820 CG5819
CG1124 CG2016
CG4322 CG4313
CG8502 CG8505
CG5171 CG5177
CG15095 CG15094
CG1915 CG1140
CG7186 CG7597
CG10462 CG10366
CG33133 CG10543
CG11670 CG11668
CG12653 CG1343
CG1213 CG1208
CG5486 CG5505
CG4914 CG7924
CG9171 CG11149
CG6824 CG32767
CG8595 CG11099
CG4746 CG4766
CG9366 CG9375
CG7526 CG32373
CG1102 CG14642
CG11824 CG8170
CG10026 CG10237
CG17359 CG17361
CG15002 CG1299 CG14990
CG8034 CG8062 CG8051
CG11228 CG10023 CG8201
CG1264 CG1046 CG1034 CG1028 CG1048
