# Supplementary material for: Selective maintenance of Drosophila tandemly arranged duplicated genes during evolution
Source: Genome Biol. 2008 Dec 16;9(12):R176. doi: 10.1186/gb-2008-9-12-r176 (PMC2646280; doi:10.1186/gb-2008-9-12-r176)
Supplement: Additional data file 9 — Groups of genes in conserved linkage between D. melanogaster and A. gambiae that are not tandem duplicates used in the embryonic co-expression analysis. [file gb-2008-9-12-r176-S9.pdf]

Additional Table 6

Groups of genes in conserved linkage between *D. melanogaster* and *A. gambiae* that are not tandem duplicates used in the embryonic co-expression analysis

1. Groups showing co-expression

nº co-expressed genes /nº genes in group*	site of co-expression	*genes in group for which in situ hybridization in the early embryo has been performed
2/2	midgut	CG7266 CG7841
2/2	midgut	CG12238 CG14222
2/2	anal pad, pharynx	CG10002 CG9990
2/2	anterior midgut	CG9300 CG9231
2/2	foregut, hindgut	CG17271 CG17272
2/3	midgut	CG7129 CG18600 CG8064
2/3	midgut	CG1910 CG2245 CG2150
2/3	midgut	CG6769 CG8142 CG6474
2/3	midgut	CG6463 CG6404 CG32067
2/3	midgut	CG5645 CG9762 CG5638
2/3	midgut	CG1753 CG1742 CG32512
2/3	visceral muscle	CG5595 CG5588 CG5596
2/4	midgut	CG13850 CG13852 CG13848 CG13849
2/4	midgut	CG1771 CG1591 CG4332 CG12096
2/4	midgut	CG8443 CG8445 CG8440 CG8441
2/4	anterior midgut	CG8625 CG8646 CG8632 CG8785
3/4	midgut	CG9175 CG13993 CG9140 CG9154
3/4	midgut	CG10535 CG17358 CG14715 CG14721
3/4	midgut	CG3297 CG13466 CG5114 CG17166
3/5	midgut	CG4299 CG4307 CG6476 CG6363 CG6623
2/6	anal pad	CG4675 CG13784 CG5160 CG4567 CG5125 CG4502
2/7	midgut	CG31043 CG5452 CG5454 CG6009 CG6013 CG6011 CG5451
2/7	midgut	CG18250 CG8314 CG8386 CG8416 CG8320 CG8418 CG8405

2/7	anal pad	CG13624 CG13633 CG17780 CG13618 CG10693 CG6593 CG5796
3/7	midgut	CG18408 CG33183 CG1371 CG1381 CG1407 CG4001 CG12920
2/8	midgut	CG3424 CG32037 CG4035 CG3431 CG3408 CG3434 CG4080 CG3654
2/8	midgut, muscle, ventrolateral sensory system	CG5461 CG17218 CG5442 CG5446 CG5525 CG5435 CG6388 CG6405
5/8	midgut	CG3409 CG3265 CG3403 CG3283 CG3268 CG3287 CG3420 CG3427
3/9	midgut	CG2666 CG32464 CG12746 CG2663 CG2161 CG2669 CG12005 CG14670 CG1172
4/9	tracheal system	CG10287 CG15188 CG1157 CG1148 CG15598 CG1169 CG1150 CG1147 CG10303
2/10	midgut, sensory system	CG14026 CG14039 CG14041 CG3792 CG8849 CG18174 CG8891 CG31660 CG3887 CG9124
3/10	midgut	CG17492 CG10689 CG10446 CG10699 CG10449 CG10691 CG10655 CG10653 CG15173 CG10563
4/10	midgut	CG31607 CG7149 CG8486 CG31605 CG7109 CG8552 CG7111 CG14536 CG7102 CG7221
2/11	midgut	CG10596 CG5249 CG17334 CG4769 CG10541 CG10640 CG4623 CG10626 CG13704 CG10672 CG7499
3/11	midgut	CG10627 CG10686 CG10698 CG10660 CG10682 CG10426 CG10646 CG4153
2/12	midgut	CG6822 CG32354 CG5989 CG32029 CG7062 CG6765 CG6745 CG6416 CG7207 CG7201 CG5962 CG7037
4/13	midgut	CG9704 CG9710 CG9706 CG9705 CG9677 CG4169 CG3971 CG9712 CG4097 CG4086 CG4109 CG13024 CG3849

2. Groups with no evidence for co-expression

**Genes in group for which in situ hybridization in the early embryo has been performed**
CG1471 CG2139
CG15669 CG9847
CG2718 CG2674
CG31431 CG6569
CG7724 CG7725
CG8583 CG8593
CG32549 CG6106
CG7758 CG10512
CG13424 CG13432
CG10207 CG10212

CG7870 CG7818
CG7720 CG12345
CG4978 CG4999
CG1441 CG2346
CG9670 CG9614
CG1119 CG31536
CG7470 CG11523
CG31175 CG31221
CG33138 CG4016
CG17124 CG6495
CG1539 CG2239
CG2679 CG16778
CG8205 CG8207
CG9322 CG9297
CG1486 CG1685
CG13209 CG12390
CG4082 CG14683
CG14655 CG17735
CG13606 CG6198
CG12026 CG13937
CG7970 CG7977
CG11966 CG11967
CG31264 CG31369
CG7839 CG6272
CG31108 CG13650
CG3962 CG5184
CG3246 CG12795
CG4654 CG4643
CG9972 CG1066
CG8733 CG8548
CG3664 CG9886
CG32982 CG9586
CG5946 CG7283
CG3860 CG3105

CG15887 CG8449
CG3520 CG3530
CG8740 CG8739
CG8603 CG8553
CG17600 CG17598
CG6619 CG6610
CG33174 CG10997
CG6767 CG8284
CG18809 CG12534
CG11326 CG17378
CG6438 CG6420
CG6199 CG32076
CG11271 CG11268
CG6625 CG6597
CG9149 CG2277
CG32372 CG7546
CG3522 CG13588
CG7807 CG32438
CG8965 CG7238
CG14968 CG17737
CG31216 CG6195
CG5502 CG4980
CG9393 CG8228
CG11660 CG7339
CG10362 CG2471
CG12015 CG3279
CG11989 CG6674
CG12220 CG12114
CG11710 CG1692
CG1599 CG1665
CG12107 CG30496
CG6287 CG6320
CG14704 CG5276
CG2184 CG15529 CG31028

CG5545 CG6433 CG6412
CG7997 CG8048 CG4311
CG9984 CG4239 CG4394
CG11661 CG3764 CG9665
CG3497 CG3994 CG15270
CG1200 CG13913 CG9122
CG1152 CG10047 CG10050
CG14447 CG5966 CG5928
CG7005 CG7012 CG7006
CG7011 CG6869 CG6876
CG8759 CG8766 CG12373
CG33323 CG10068 CG31248
CG13969 CG10756 CG10757
CG11128 CG11115 CG10712
CG31323 CG14543 CG6154
CG9351 CG9374 CG14367
CG31757 CG14940 CG14939
CG3910 CG3909 CG11722
CG6459 CG30105 CG10933
CG7399 CG8492 CG7391
CG18654 CG14764 CG14763
CG11561 CG11617 CG3645
CG5248 CG6949 CG6958
CG5641 CG5266 CG5844
CG1782 CG1776 CG18345
CG1759 CG9318 CG9324
CG17265 CG3542 CG17258
CG9554 CG9526 CG9542
CG7920 CG7921 CG7903
CG2078 CG1968 CG1975
CG8938 CG6953 CG6984
CG12230 CG14217 CG33525
CG30427 CG2765 CG3760
CG5422 CG33100 CG6147

CG10938 CG4798 CG6550
CG30118 CG5186 CG5190
CG4778 CG4839 CG4758
CG11136 CG8994 CG11132
CG6965 CG31368 CG14724
CG8024 CG8026 CG8055
CG8912 CG5935 CG8905
CG11887 CG12338 CG12341
CG1344 CG10465 CG10417 CG10395
CG5064 CG5068 CG5651 CG4911
CG7265 CG3731 CG7281 CG33332
CG3510 CG30092 CG9952 CG30271
CG12758 CG15097 CG15084 CG15081
CG31670 CG7337 CG31665 CG31663
CG4133 CG4063 CG4164 CG4033
CG11241 CG11370 CG11367 CG6838
CG11295 CG5472 CG5411 CG9850
CG6281 CG3999 CG6217 CG6293
CG13037 CG16807 CG4672 CG4561
CG3048 CG4104 CG2960 CG3702
CG9653 CG9657 CG1643 CG8300
CG1440 CG11202 CG2151 CG10964
CG2054 CG13922 CG13921 CG12023
CG1225 CG32344 CG32343 CG7008
CG5130 CG6020 CG5955 CG5047
CG3938 CG3903 CG4152 CG13240
CG6188 CG6174 CG14395 CG17369 CG6019
CG10371 CG10230 CG10370 CG10221 CG10365
CG13387 CG13391 CG17292 CG13390 CG13398
CG3074 CG10382 CG5799 CG3425 CG3045
CG4710 CG4385 CG4552 CG5041 CG4749
CG3722 CG15651 CG30295 CG10540 CG9344
CG11024 CG14021 CG7382 CG14022 CG7371
CG14830 CG8615 CG8614 CG8610 CG8616

CG3385 CG3376 CG3401 CG3394 CG3318 CG30418
CG1897 CG14509 CG14516 CG11500 CG14507 CG11899
CG17914 CG17332 CG17905 CG31991 CG13277 CG31739
CG30023 CG13196 CG8991 CG7776 CG8238 CG8998
CG9768 CG9779 CG1074 CG1090 CG14647 CG9766
CG9256 CG1099 CG1762 CG9250 CG9339 CG9265
CG7891 CG2708 CG10903 CG2767 CG9667 CG11035
CG16984 CG32300 CG9018 CG8993 CG5714 CG12187
CG3331 CG5802 CG3353 CG6056 CG5919 CG5862 CG5745
CG1624 CG11140 CG11139 CG11127 CG30499 CG1669 CG30492
CG15162 CG10275 CG10338 CG10346 CG10283 CG15160 CG10343
CG4532 CG3184 CG3960 CG3135 CG3973 CG14444 CG3126 CG12796
CG15009 CG18675 CG14996 CG1308 CG14994 CG14993 CG1318 CG14995 CG1319 CG15005 CG15011 CG1309 CG11586
CG10072 CG10160 CG10064 CG9953 CG10118 CG10173 CG10107 CG32392 CG8398 CG10060 CG18769 CG33171 CG32393 CG8368 CG8631
