# Supplementary material for: Selective maintenance of Drosophila tandemly arranged duplicated genes during evolution
Source: Genome Biol. 2008 Dec 16;9(12):R176. doi: 10.1186/gb-2008-9-12-r176 (PMC2646280; doi:10.1186/gb-2008-9-12-r176)
Supplement: Additional data file 10 — Stringency in the definition of conserved TDGs as used in this study. [file gb-2008-9-12-r176-S10.pdf]

Additional Note

We have been extremely careful in the scoring of the in situ data, and have used extremely conservative criteria for assigning positive evidence for co-expression. In first place, all the analysis was performed using Ensembl gene identifiers (CGs), and only later were known gene names added, in order to avoid a prior assumptions about common expression. For instance, both the ANTP and BX Hox complexes were among the set of conserved TDGs that had available in situ evidence. Genes from the ANTP complex (*lab*, *pb*, *scr* and *Antp*) show sequential expression along the anterior segments, what involves conserved coordinated cis-regulatory mechanisms. However, we did not score these genes as positive because there is no overlap between the expression domains of the different genes. In the case of *Ubx* and *abd-A* from the BX complex, we did score them as positive because the expression domain are overlapping in posterior segments [see Additional data file 11].

Furthermore, we are aware that because of the stringent phylogenetic criteria followed to define a group of tandem duplicates as evolutionarily conserved (that the number of inter-species similarities outscores those between peptides from the same specie; see Materials and Methods) a number of cases will have not been included in our conserved TDG group.

Among these, and as an example, we show below the results for the *dorsocross* T-box transcription factor family. There are three *Doc* genes in *D. melanogaster* arranged in tandem [32, 50], and two in *A. gambiae* in tandem as well. Alignment of the predicted peptides show a high degree of aminoacid conservation (Fig. S1), and all three *D. melanogaster* genes are co-expressed in the early embryo (Fig. S2). Furthermore, recent evidence shows that both *A. gambiae* genes are expressed in an equivalent domain in the mosquito embryo [51]. However, when we construct the phylogenetic tree for the five peptides, *A. gambiae* Doc1 and Doc2 group independently from the *D. melanogaster* peptides (Fig. S3). This topology would be rejected by our methods and therefore, the *Doc* gene family would not be considered as a conserved TDG. Although it can be argued that *Doc* genes duplicated independently in the *D. melanogaster* and *A. gambiae* lineages, this does not seem very probable and we suspect that tree topology is caused by divergence rate of the different peptides. We find the same situation for other genes families, such as the *Iroquois* complex [6], which is formed by three homeobox genes in *D.*

melanogaster and two linked genes in *A. gambiae* (data not shown). In light of these cases, we believe that our recount of cases of conserved TDGs that are co-expressed in the embryo is clearly an underestimate, and that the rate of false positives (two linked duplicated genes that are co-expressed by chance) will be very low, if not negligible.

One final observation is that among the patterns shown by co-expressed genes we found a high proportion that corresponds to the embryonic midgut. Out of 58 groups of non conserved TDGs that were co-expressed [see Additional data file 6], 16 were so in the midgut. In the case of conserved non TDGs, it is 11 out of 38 co-expressing groups, and a staggering 28 out of 37 co-expressing groups of conserved neighbours do so in the midgut. Surprisingly, none of the conserved TDGs that were co-expressed corresponds to midgut expression. This might indicate that this is a very common site of expression in the embryo, and therefore we could be finding instances of co-expression that were due to chance. In any case, such possibility would only mean that our observation that conserved TDGs tend to show a higher degree of co-expression would be even more significant.

```
                  10        20        30        40        50        60        70        80        90
         ....|....|....|....|....|....|....|....|....|....|....|....|....|....|....|....|....|....|
Dm Doc1  MLSMQELMDMRMQQQLAH--EIYRQQIMQRIPDPFPSMLPFNMPHQQPHMMLPSRPTLPG-VEAKLENNDLWQQFHKIGTEMIITKSGRR
Dm Doc2  MITMNELVDLRMQQHIAH--EIYRQQIMQRIPDPFPPMLPIPMP--RHVIMPPRV-TLPG-VEMTLQNDDLWKQFHQIGTEMIITKSGRR
Dm Doc3  MLTLPNIADLRLQQQIAQ--EIYRQQIMQRLPDPLCGLLPNFAGHFMPPPPPPQP-TLPGDVEAKLENNELWQQFHSIGTEMIITKCGRR
Ag Doc1  -TTMTDLIDLRMHQHIAH--EIYRQQMLQRIPDPFPTMLPVPVPPMHAMALPPRY-SLPG-VELKLQNKELWREFHKIGTEMIITKSGRR
Ag Doc2  --MTADLMDLRMHHHLALQTEFYRMQ-MQRLPDPYPAMLPVPAP--RPMMIPPRYNALPD-VDIKLQNRDLWSQFHKIGTEMIITKSGRR

                 100       110       120       130       140       150       160       170       180
         ....|....|....|....|....|....|....|....|....|....|....|....|....|....|....|....|....|....|
Dm Doc1  MFPSMRVSLSGLEEEASYCVLLEMVPIGDCRYKFSGSQWVPAGGAEPQSPQRMYLHPDSPATGAHWQSQALLFNKVKLTNNTLDSSGHIV
Dm Doc2  MFPSMRLSVSGLEDESNYCVLLEMVPIGDCRYKFSGSQWVPAGGAEPQSPQRMYLHPDSPATGAHWQAQPILFNKVKLTNNTLDNSGHIV
Dm Doc3  MFPSMRVSLSGLEEEASYCVLLEMVPIGDCRYKFSGSQWVPAGGAEPQSPQRMYLHPESPATGKHWQSQALLFSKVKLTNNTLDNNGHIV
Ag Doc1  MFPSMRLTVDGLDADTNYCVLLEMMPISDCRFKFSGSQWVPAGGAEPQSPQRFCLHPDSPALGTHWASQPIVFNKVKLTNNTLDNNGHVV
Ag Doc2  MFPSMRLSVNGLEAEENYFILLEMVPISDCRFKFSGSQWVPAGGAEPQSPQRIYFHPDGPALGSHWTSQPIVFNKVKLTNNTLDSNGHIV

                 190       200       210       220       230       240       250       260       270
         ....|....|....|....|....|....|....|....|....|....|....|....|....|....|....|....|....|....|
Dm Doc1  LASMHKYQPRLHIIRSSELTQLPWAPQQAFVFPETEFVAVTAYQNDRITKLKIDNNPFAKGFRESGQSRCKRKLNSSGNS----------
Dm Doc2  LASMHKYQPRLHVIRTADLAQIPWAPQQAFVFAETEFVAVTAYQNDRITKLKIDNNPFAKGFRETGQSRCKRKMSSSPTGEDQDQSPSLS
Dm Doc3  LASMHKYQPRLHVIRTSDLGQIPWAPQQAFIFPETEFIAVTAYQNDRITKLKIDNNPFAKGFRESGQSRCKRKINDSPPP----------
Ag Doc1  LTSMHKYQPRIHIIRTSDPSQIPWAAQQAFVFPETEFVAVTAYQNDRITKLKIDNNPFAKGFRETGQSRCKRKMGCNNTS---------S
Ag Doc2  LTSMHKYQPRIHVIKASDATQIPWAPQQAFTFPETEFVAVTAYQNDRITKLKIDNNPFAKGFRETGQSRCKRK-----------------
```

Figure S1. Alignment of Doc predicted peptides from *D. melanogaster* (*Dm*) and *A. gambiae* (*Ag*). Positions where at least four out of five residues are identical are highlighted. The last residues from *D. melanogaster* proteins are not shown.

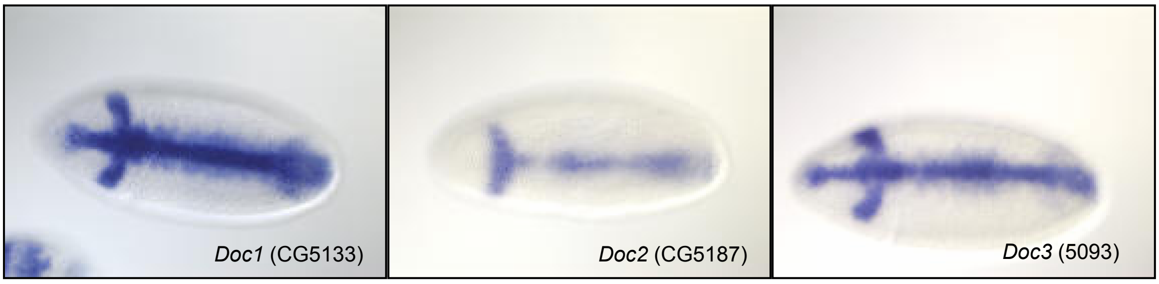

Figure S2. Expression pattern of the three *D. melanogaster Doc* genes at early stages. Co-expression in the dorsal midline and anterior cephalic region is evident.

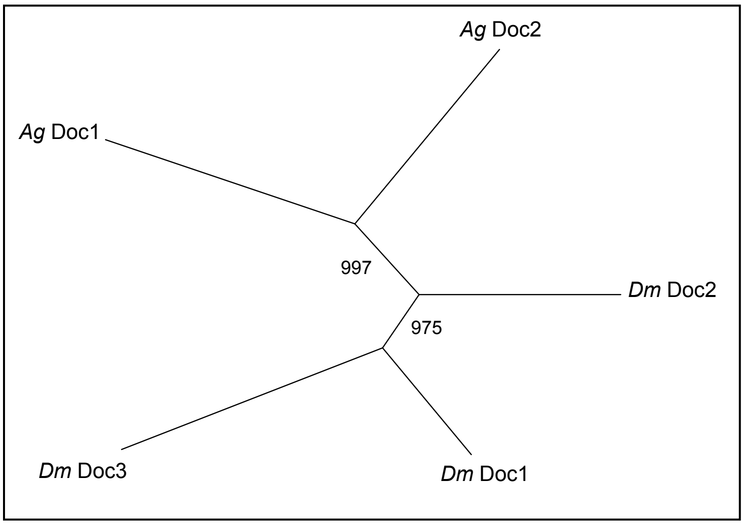

Figure S3. Unrooted NJ phylogenetic tree of predicted Doc peptides, showing bootstrap values for 1000 replicates. *A. gambiae* peptides group independently from *D. melanogaster* peptides, but nevertheless branch length is high in all lineages.
