# Supplementary material for: Selective maintenance of Drosophila tandemly arranged duplicated genes during evolution
Source: Genome Biol. 2008 Dec 16;9(12):R176. doi: 10.1186/gb-2008-9-12-r176 (PMC2646280; doi:10.1186/gb-2008-9-12-r176)
Supplement: Additional data file 11 — Conserved TDGs that are co-expressed in the Drosophila embryo that have been previously described in the literature. [file gb-2008-9-12-r176-S11.pdf]

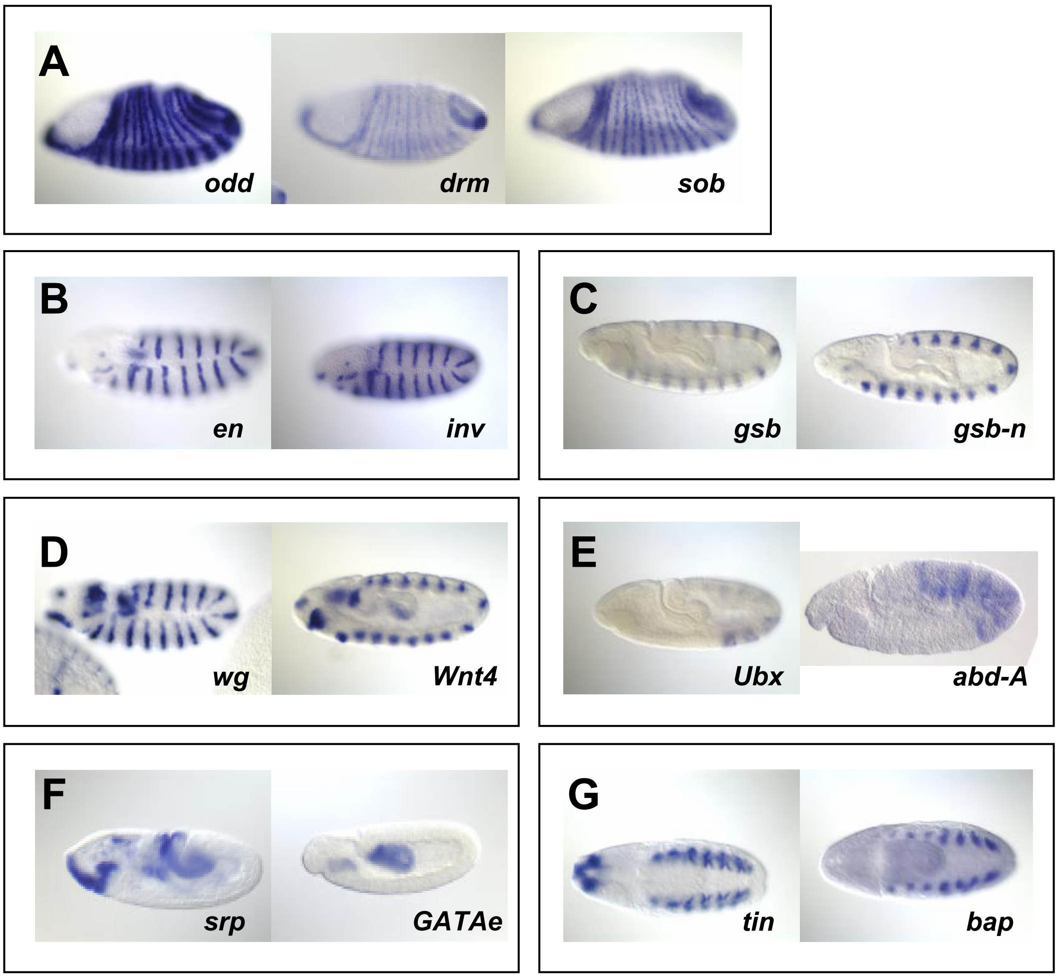

Additional Figure 4. Conserved TDGs showing co-expression in the *Drosophila* embryo that have been previously described. These groups were identified in our analysis with no prior knowledge about their identity. Among them are groups of homeobox genes, such as the segment polarity genes *en*/*inv* (B) and *gsb*/*gsb-n* (C), the Hox genes *Ubx* and *abd-A* (E), and the muscle specifiers *tin*/*bap* (G); the zinc fingers transcription factors *odd*/*drm*/*sob* (A) and *srp*/*GATAe* (F); and the signalling factors *wg*/*Wnt4* (D). See main text for references.
